# Comparative Analysis of Seizure Clusters in Patients with and Without a History of Epilepsy Presenting to the Emergency Department

**DOI:** 10.3390/neurosci6030079

**Published:** 2025-08-13

**Authors:** Silvio Basic, Ivana Basic, Ivana Susak Sporis, Davor Sporis, Jelena Saric Juric, Petra Meznaric

**Affiliations:** 1 Department of Neurology, University Hospital Dubrava, 10000 Zagreb, Croatia; sbasic@kbd.hr (S.B.);; 2 School of Medicine, J.J. Strossmayer University of Osijek, 31000 Osijek, Croatia; 3 School of Medicine, University of Zagreb, 10000 Zagreb, Croatia; 4 Department of Neurology, Neurosurgery and Neuropathology, University of Applied Health Sciences, 10000 Zagreb, Croatia; 5 Department of Neurology, University Hospital Centre Osijek, 31000 Osijek, Croatia

**Keywords:** seizure, cluster, epilepsy, emergency department, risk factor

## Abstract

Seizure clusters can be observed in patients with epilepsy as well as in individuals without a previous history of epilepsy. However, there are no data on whether seizure clusters differ between these two populations. The purpose of this study was to investigate the clinical presentation, diagnostic findings, presence of seizure triggers, outcomes and complications of seizure clusters in patients with epilepsy and individuals without epilepsy in their medical history. The results indicate that epilepsy history was not independently associated with the number of seizures during cluster; however, increasing age was significantly associated with a lower seizure burden, and pneumonia demonstrated a marginal positive association. Structural brain lesions were prevalent in both groups; particularly chronic post-stroke lesions and frontal lobe lesions were significantly more common among epilepsy patients. Over half of patients without prior epilepsy received a new epilepsy diagnosis following the cluster event. No severe complications, including status epilepticus or postictal psychosis, were observed. Our findings suggest that age, acute comorbidities, and structural brain pathology likely exert greater influence on frequency of seizures during cluster. Chronic post-stroke lesions, which have not yet been reported as a risk factor for seizure clusters, were the most frequent brain pathology in both groups and may thus be considered as an additional risk factor for this clinical entity. Prospective and larger-scale studies are needed to further clarify these associations.

## 1. Introduction

Seizure clusters—also called repetitive, serial, crescendo, acute repetitive, and recurrent seizures—are a clinical phenomenon characterized by grouped seizures with short interictal periods [1,2]. Unfortunately, there is still no consensus in terms of the definition of seizure clusters [3]. Previous studies have defined seizure clusters differently regarding the number of seizures and cluster duration, including as ≥2 seizures within 6 h, ≥3 seizures within 24 h, and ≥2 seizures within 48 h [4,5,6,7,8,9,10]. Recent consensus recommendations provided by the International League Against Epilepsy (ILAE) expert group rejected a specific time-delimited definition of seizure clusters and proposed the definition of seizure clusters based on comparison with the patient’s usual seizure pattern [11].

Regardless of the definition, all studies indicated more or less the same risk factors for seizure clusters. Frontal lobe epilepsy, mesial temporal sclerosis, multifocal epilepsy, symptomatic generalized epilepsy, remote history of central nervous system (CNS) infection, and focal cortical dysplasia have all been identified as risk factors for seizure clustering [7,8,12,13]. Moreover, status epilepticus, history of seizure clusters, earlier age of seizure onset, high seizure frequency, and head trauma have also been associated with seizure clusters [6,8,12,13]. Chen et al. observed that patients with structural generalized epilepsy are more likely to experience seizure clusters than patients with epilepsy of other etiology [8]. Several acute precipitating factors for seizure clustering have been reported, including sleep deprivation, stress, fever, acute illness, missed or changing medications, alcohol consumption, and menstruation [3]. However, seizure clusters can occur in patients without identifiable risk factors.

Seizure clusters have been associated with a high rate of hospital admissions, an risk of developing status epilepticus if left untreated, seizure-related injuries, higher mortality rates, and an increased risk of postictal psychosis [2,3]. The most important adverse outcome is progression to status epilepticus, which is a life-threatening condition that requires prompt medical intervention. According to the recent Consensus recommendations, rapid and early seizure termination (REST) medication or acute cluster treatment (ACT) should be considered as soon as the SC pattern is recognized, and in patients who have seizure clusters as their main seizure pattern, treatment should be considered at the onset of the first seizure, in order to prevent potential complications [11]. Most studies on seizure clusters have been conducted in tertiary epilepsy centers with selected groups of patients, frequently patients with pharmacoresistant epilepsy. However, seizure clusters may also be observed in patients without a previous history of epilepsy or isolated epileptic seizures. The data regarding seizure clusters in such patients are scarce.

The purpose of this study was to investigate the causes, presentation, and outcomes of seizure clusters in patients admitted to the emergency department (ED). Moreover, we aimed to determine whether there was a difference in seizure clusters between patients with epilepsy and those without epilepsy in their medical history.

## 2. Patients and Methods

This study was a single-center retrospective study designed to determine the prevalence, causes, and outcomes of seizure clusters in subjects admitted to our ER during a period of 5 consecutive years. For the purpose of this study, seizure clusters were defined as the occurrence of two or more epileptic seizures within a 24 h period in adults or within a 12 h period in children, consistent with previously reported definitions [10]. Although the recent consensus recommendations from ILAE expert group reject a strict time-delimited definition of seizure clusters, the proposed definition in not applicable in patients without previous history of epilepsy [11]. To maintain consistency across all study subjects, irrespective of epilepsy history, we applied the operational definition of seizure clusters as two or more epileptic seizures within 24 h as our main inclusion criterion. Exclusion criteria comprised psychogenic non-epileptic seizures, status epilepticus, syncope or transient loss of consciousness due to non-epileptic causes and other seizure mimics.

This study was conducted as a retrospective chart review using data extracted from the electronic medical records (EMR) system of our hospital. To ensure privacy and confidentiality, all patient data were fully anonymized prior to the analysis, with unique identifiers removed or replaced.

The following variables were recorded for each patient: demographic characteristics (age, sex), epilepsy history (presence or absence of a prior epilepsy diagnosis, etiology of epilepsy, type of seizures, seizure control status and adherence to antiseizure medicines), seizure cluster characteristics (total number of seizures during the cluster episode, type of seizures observed), precipitating factors (sleep deprivation, emotional stress, alcohol or substance abuse, recent infections, or missed ASM doses), prior brain insult (stroke, perinatal brain injury), diagnostic findings (laboratory parameters, neuroimaging findings and EEG), treatment and outcomes (therapies administered in the ED, need for hospital admission, and clinical outcomes during ED stay and hospitalization).

To enable comparison, patients were divided into two groups based on their history of epilepsy: those with epilepsy and those without.

Data analysis was performed with the SPSS statistical analysis software, version 20.0 (IBM Corp., Armonk, NY, USA). Appropriate tests were performed based on the dataset. Differences in the data were considered statistically significant at *p* < 0.05.

## 3. Results

The overall period prevalence of seizure clusters was 6.41 per 10,000 (CI, 4.76–8.45). In patients without epilepsy, the prevalence rate was 2.05 per 10,000 (CI, 1.19–3.31); in patients with epilepsy, it was 1.3 per 10 (CI, 0.96–1.93). During the defined study period, a total of 47 patients fulfilled the inclusion criteria, with a higher proportion of men (*n* = 32, 68%) than women (*n* = 15, 32%). The overall median age was 49.5 years (IQR 28.5), with no statistically significant difference in age between the two groups; however, patients without epilepsy tended to be older. There were 32 (68%) patients with epilepsy and 15 patients (32%) without epilepsy in their medical history (Table 1).

**Figure 1:**
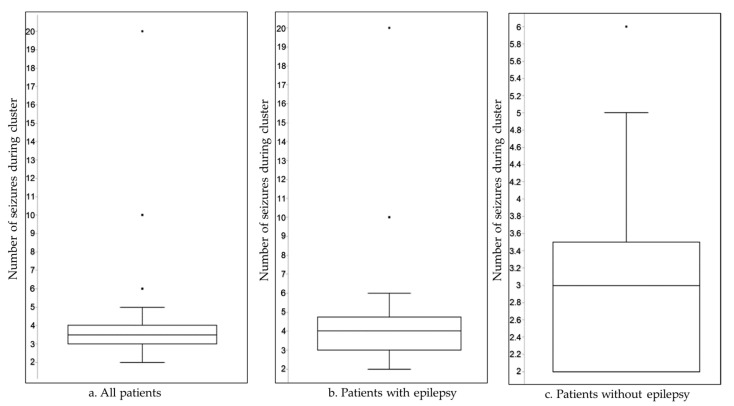
Median number of seizures (IQR): (**a**) all patients, 3.5 (1); (**b**) patients with epilepsy, 4 (1.75); (**c**) patients without epilepsy, 3 (1.5). Mann–Whitney U test, U = 81, Z = 2.37016; *p* = 0.017.

The number of seizures per cluster ranged from 2 to 20 within 24 h, with a higher seizure count during clusters in patients with epilepsy (Figure 1).

In a multivariable Poisson regression model adjusting for age, sex, comorbidities (urinary tract infection, pneumonia), and etiology (tumor, stroke, trauma, PN brain injury), epilepsy status was not independently associated with number of seizures during cluster (IRR = 1.05; 95% CI: 0.65–1.69; *p* = 0.853). In contrast, increasing age was significantly associated with a lower seizure burden (IRR = 0.98; 95% CI: 0.97–0.99; *p* = 0.004). Other covariates, including sex and comorbid conditions, were not significantly associated with the number of seizures during cluster (Table 2).

Generalized tonic–clonic seizures (GTCS) were the most frequently observed seizure type in both groups. In patients without epilepsy, these were followed by focal impaired awareness seizures to bilateral tonic–clonic (FIAS to BTC) in the group of patients with epilepsy, they were followed equally by focal aware seizures (FAS) and focal aware seizures to bilateral tonic–clonic (FAS to BTC). Fisher’s exact test revealed no statistically significant differences in seizure type distribution between patients with epilepsy (WE) and those without epilepsy (WOE) after correcting for multiple comparisons. While FAS to BTC seizures were observed exclusively in the epilepsy group this difference was not statistically significant (uncorrected *p* = 0.162; Bonferroni-adjusted *p* = 0.809; FDR-adjusted *p* = 0.404). Conversely, FIAS to BTC seizures were more frequent in the WOE group yielding a borderline significant result (*p* = 0.089); however, this trend was also no longer significant after correction (uncorrected *p* = 0.089; Bonferroni-adjusted *p* = 0.446) (Table 3).

Comparison of seizure types between patient history and documented seizure clusters revealed no statistically significant differences. GTCS were more frequently observed during clusters (56%) compared to historical reports (37%), but this difference was not significant (*p* = 0.21). Similarly, FAS and FIAS occurred slightly more often during clusters than in history, though differences were not statistically meaningful. FIAS to BTC seizures appeared more often in history than during clusters (16% vs. 3%), yielding a non-significant but notable odds ratio of 5.74 (*p* = 0.20). Overall, no seizure type showed a statistically significant shift between history and cluster periods (Table 4).

One third of epilepsy patients had poor seizure control in their medical history, and non-compliance was observed in 12.5%. The median for epilepsy duration was 5.5 years (IQR 16.25). The etiology of epilepsy was dominantly structural (56.3%) followed by unknown (25%), genetic (12.5%), and metabolic/toxic (6.2%). The most frequent pathology in patients with structural epilepsy was remote cerebrovascular disorder (i.e., ischemic stroke or intracerebral hemorrhage), which was present in 50% of patients followed by perinatal brain damage (22.2%), brain trauma (16.7%), and brain tumor (11.1%). In patients without a previous history of epilepsy, toxic etiology (chronic alcohol abuse) was as frequent as structural brain changes detected by neuroimaging performed in the ED. Remote cerebrovascular disorder was also a leading brain pathology. There was no significant difference in the proportion of brain lesions between the two groups of patients (Z = 0.3466, *p* = 0.363). The patients with epilepsy had a statistically higher frequency of frontal lobe lesions (72.7%) than patients without epilepsy (Fisher’s exact test, *p* = 0.0256), while there was no difference regarding other localizations. An acute infection was diagnosed in 27.6% cases, of whom 84.6% were patients with epilepsy. Regarding other laboratory findings, no abnormalities were observed, except for hyponatremia in three patients. In all cases, sodium levels ranged between 125 and 129 mmol/L. Two of these patients had epilepsy and were taking oxcarbazepine, while the third was a patient without epilepsy who had chronic polydipsia. Chronic daily use of alcohol was observed in 17.02% of patients, with a higher frequency in patients without a history of epilepsy. Among the patients without a previous history of epilepsy, we identified three patients with no risk factors or comorbidities to explain their seizure clusters. In those patients, the seizure cluster was the first presentation of epilepsy, which was later classified as of unknown etiology during follow-up. Fisher’s exact tests were used to compare EEG findings between epilepsy and non-epilepsy patients. Normal EEGs were seen in 6/32 epilepsy patients versus 6/15 non-epilepsy patients (OR = 0.47, *p* = 0.31). Generalized discharges were present in 10 vs. 6 patients (OR = 0.78, *p* = 0.74), and focal discharges in 16 vs. 3 patients (OR = 1.72, *p* = 0.39). No differences were statistically significant, suggesting that the distribution of these EEG findings did not differ between groups.

Intravenous diazepam was successfully administered to 19.1% of patients in the ED. A repeated seizure cluster (within the next 48 h) was observed in only one patient. Postictal psychosis and status epilepticus were not observed in this study. At discharge, epilepsy was diagnosed in 53% of patients without a previous history of epilepsy, according to the practical clinical definition of epilepsy [14]. A total of five patients were admitted to the hospital. Among them, two had a known history of epilepsy—one was admitted due to an acute femoral fracture requiring surgical intervention, and the other due to repeated episodes of acute repetitive seizures (ARS). The remaining three patients did not have epilepsy: two were admitted for complicated urinary tract infections, and one due to the newly diagnosed brain tumor.

## 4. Discussion

In this study, we aimed to determine the characteristics, etiologies, and clinical outcomes of seizure clusters in both patients with epilepsy and those without epilepsy in their history. Moreover, our primary aim was to determine whether there is a difference in seizure clusters between those two groups, as data regarding seizure clusters in patients without epilepsy in their medical history are scarce. The inclusion of patients without prior epilepsy highlights the diagnostic and prognostic role of seizure clusters in identifying individuals at high risk for subsequent unprovoked seizures—a central tenet in the International League Against Epilepsy (ILAE)’s revised practical definition of epilepsy [14].

In our cohort of patients attending the ED, the overall period prevalence of seizure clusters (6.41 per 10,000; CI, 4.76–8.45) was similar to previously reported estimated data, indicating that seizure clusters comprise approximately 10–20% of epilepsy-related ED attendances [2,15,16]. Regarding demographics, we observed a higher proportion of male patients with a median age of 49.5 years and no significant age difference between the two observed groups. These data are concordant with the previously reported higher incidence of seizure clusters and acute symptomatic seizures in adult males [17,18]. This may be due to the higher incidence of comorbid conditions in this population, which could potentially act as provoking or risk factors for seizure clusters. A higher number of seizures per cluster was observed in patients with epilepsy; however, this difference did not reach statistical significance. This could reflect poor seizure control or non-adherence to therapy prior to clusters; however, in our study, only one-third of patients had poor seizure control, and an even smaller proportion were non-adherent to their standard therapy. In the multivariable Poisson regression analysis adjusting for age, sex, comorbidities, and etiology, epilepsy was not independently associated with seizure cluster frequency (IRR = 1.05; 95% CI: 0.65–1.69; *p* = 0.853). Increasing age remained significantly associated with lower seizure counts (IRR = 0.98; 95% CI: 0.97–0.99; *p* = 0.004), suggesting that seizure burden decreases with advancing age. Notably, the presence of pneumonia was associated with a 52% increase in seizure rate (IRR = 1.52; 95% CI: 0.98–2.34; *p* = 0.059), indicating a marginal association that may reach significance in larger cohorts or in specific clinical contexts. Other variables, including sex, UTI, remote stroke, trauma, PN brain injury, and tumor, were not significantly associated with seizure cluster count.

The observed seizure semiology indicates the variable nature of seizure clusters. In both groups, GTCS were the most frequently observed seizure type, with differences observed in the prevalence of other types of seizures. In epilepsy patients, FAS and FAS to BTC were more common; this could be a reflection of structural brain lesions, which was the etiology with the highest prevalence in this group of patients. Similarly, in patients without a previous history of epilepsy, FIAS to BTC seizures were more common. Most patients in this group had extrafrontal brain lesions. FAS to BTC seizures were observed exclusively in the epilepsy group while FIAS to BTC were more frequent in patients without previous history of epilepsy (3 vs. 1 case), yielding a borderline significant result (*p* = 0.089) that may indicate a potential trend that warrants further investigation.

When considering etiology, structural brain abnormalities were the most frequent comorbidity in this study in both groups. In patients with epilepsy, structural etiology was present in more than half of patients (56.3%). The etiological analysis revealed that remote cerebrovascular insults such as ischemic stroke or intracerebral hemorrhage were the most frequent causes of structural epilepsy. This finding aligns with the literature identifying cerebrovascular disease as a leading cause of both late-onset epilepsy and seizure clusters in the elderly [19]. Interestingly, the same lesions were also the most common etiology in patients without a previous history of epilepsy. In this case, seizure clusters were the primary manifestation of ongoing epileptogenesis in the setting of a pre-existing previously silent brain injury. Hence, patients without epilepsy in their history were diagnosed with structural epilepsy following seizure clusters, according to the ILAE’s practical definition of epilepsy [14].

Regarding structural etiology, our findings are partially similar to the results of Haut et al., who reported an association between seizure clusters in patients with symptomatic epilepsy and a history of head trauma [6]. In the context of brain lesions, pathophysiologically, stroke and traumatic brain lesion share common secondary injury mechanisms including oxidative stress, moderate-to-high inflammation resulting in neuronal death, gliosis, and inflammation leading to hyperexcitable neuronal networks and, hence, epilepsy. Interestingly, a history of head trauma was present in less than 10% of patients in our study.

Among patients with structural epilepsy, frontal lobe damage was present in more than two thirds of patients. Additionally, patients with epilepsy exhibited a higher prevalence of frontal lobe lesions (72.7%) compared to those without epilepsy (*p* = 0.0256). This is in concordance with previous suggestions of extratemporal lobe epilepsy as a risk factor for seizure clustering [6,7]. Moreover, frontal lobe epilepsy has been frequently associated with seizure clusters due to its rich corticocortical connections and propensity for high seizure propagation potential [6,20,21]. No significant difference in the overall presence of structural brain lesions between groups was detected, indicating that clustering may occur regardless of lesion burden but might be influenced by localization. We acknowledge the presence of missing data that may have impacted the complete-ness of our analysis. CT imaging findings were unavailable for seven patients due to imaging not being obtained at the time of presentation.

We compared our results with other previously reported risk factors for seizure clusters in patients with epilepsy, including previous seizure clusters, a longer duration of epilepsy, poor seizure control, and a history of head trauma [2]. In contrast to previous reports, our study resulted in a somewhat different risk factor profile. In our group, only two patients experienced a seizure cluster previously, indicating that this may not be a prevalent risk factor for seizure clusters. Moreover, almost half of the epilepsy patients in our cohort had an epilepsy duration of less than five years, which is in contrast with a previous report where a longer epilepsy duration was often associated with an increased likelihood of seizure clusters [22]. Furthermore, only one third of the patients in our study had previous poor seizure control, which is considered as a significant risk factor for seizure clusters. As we work in a tertiary epilepsy center dealing with pharmacoresistant patients, the number of observed clusters in poorly controlled epilepsy is significantly lower than the number of patients with poorly controlled seizures. Hence, poor seizure control appears to be an important—though not universal—risk factor for seizure clusters.

Our study suggests that the relationship between the observed risk factors and the occurrence of seizure clusters in patients with epilepsy may not be as straightforward as previously thought. However, there is a need for a more nuanced understanding of all contributors included in the onset of seizure clustering [23].

Different triggers for seizure clusters have been described thus far. They include sleep deprivation, stress, illness, fever, missing or changing medications, menstruation, and alcohol consumption [5,13]. Sleep deprivation and stress were not present in the patients included in this study. However, the data were not systematically collected and were entirely lacking in 36% of patients. Non-compliance was observed in 12.5% of patients with epilepsy, and none of the patients were in the process of therapy modification. Regarding the non-compliance, which was observed in only small proportion of our epilepsy patients, we acknowledge the limitation of non-obtaining ASM level measurements. However, such analyses are not available in our emergency setting. We acknowledge that ASM levels could provide an objective indication of adherence; however, they are not a reliable stand-alone measure. They can be a helpful tool, but only within a broader clinical context. Moreover, the epilepsy patients admitted to the ED are regularly followed in our outpatient clinic over a number of years, and we are familiar with their adherence patterns, allowing us to identify by name those who are generally adherent and those who are less reliable regarding medication adherence. An acute infection was diagnosed in 27.6% of patients, predominantly among those with epilepsy (84.6%). These results are expected since infections are well known as seizure triggers due to systemic inflammatory responses, fever, and metabolic stress. On the other hand, chronic use of alcohol was observed more frequently in patients without a previous history of epilepsy.

In our cohort, EEG findings did not differ significantly between patients with epilepsy and those without. The lack of difference in EEG patterns supports the notion that routine interictal EEG findings may not reliably indicate seizure cluster propensity. In other words, patients who experience clusters may not exhibit distinctive EEG features detectable on routine EEG.

Except for one patient who experienced a repeated seizure cluster within 48 h during their hospital stay, we did not observe any other complications, including status epilepticus and postictal psychosis. This finding should be interpreted cautiously, given the setting of the study (tertiary center) and small sample size. However, it could imply and highlight the importance of early administration of benzodiazepines as a rescue therapy in these patients, since the data from real-life studies have shown that these medications are often underutilized [24,25]. While many patients in our epilepsy cohort had prescriptions for rescue benzodiazepines, only six individuals actually administered them before seeking emergency care. Our sample likely represents a subset of individuals experiencing seizure clusters who either did not respond to rescue benzodiazepines or, more commonly, did not attempt to use them despite having access. This has important implications for interpreting severity—patients presenting to the ED may not be inherently more medically severe than others with seizure clusters but may reflect a failure or omission in timely outpatient management.

It is important to distinguish the population studied here from those typically evaluated in studies of status epilepticus. Prior studies on status epilepticus often report higher rates of ICU admission, prolonged hospital stays, and more significant morbidity [26]. In contrast, our cohort included patients with potentially self-limited events. The low use of rescue benzodiazepines prior to the ED presentation in our cohort further highlights a difference in management and possibly in severity. While both groups represent serious seizure emergencies, patients in this study likely reflect a more heterogeneous and potentially less severe group.

The diagnosis of epilepsy in more than half of patients with seizure clusters and without epilepsy in their history, indicates its clinical utility, especially in patients with comorbid conditions linked to epilepsy. Interestingly, in three patients without a previous history of epilepsy, a seizure cluster was the first presentation of epilepsy, which was classified as being of unknown cause during follow-up. This highlights the utility of seizure clusters as an indicator of underlying epilepsy and emphasizes the need for further monitoring of those patients.

Long-term follow-up of patients with seizure clusters but no initial epilepsy diagnosis will be particularly valuable in understanding the risk of developing chronic epilepsy and informing early treatment decisions.

## 5. Conclusions

Our study revealed some differences in seizure clusters between patients with epilepsy and those without a history of epilepsy. Seizure clusters were more frequent in patients with epilepsy, who also experienced higher seizure frequency during clusters. However, in multivariable analysis epilepsy status was not independently associated with seizure cluster frequency after adjusting for demographic, clinical, and etiological factors. Age emerged as a significant predictor, with older patients exhibiting lower seizure burden. Pneumonia demonstrated a marginal association with increased seizure frequency, suggesting it may contribute to seizure exacerbation in certain clinical scenarios. These findings highlight the importance of considering both age and acute comorbidities when evaluating seizure cluster risk, rather than epilepsy diagnosis alone.

All seizure types occurred at similar rates between groups, and our findings suggest no clear distinction in seizure type patterns between patients with and without epilepsy in this sample. Seizure clusters were the first symptom of underlying epilepsy in half of the patients without a previous history of epilepsy. Hence, seizure clusters in those patients should be taken seriously as they may resemble previously undiagnosed epilepsy. Regarding triggers, acute infection was a frequent comorbidity in patients with epilepsy, while chronic alcohol abuse was more frequently observed in patients without epilepsy. Frontal lobe lesions were more frequent in patients with epilepsy and seizure clusters. Remote cerebrovascular disorder—which has not yet been reported as a potential risk factor for seizure clusters—was the most frequent brain pathology in both groups.

The main limitations of this study are its retrospective design and small sample size, which reflect the observed low overall incidence of seizure clusters. Moreover, selection bias may arise because our sample consists only of patients who presented to the ED. This likely excludes individuals with epilepsy who manage seizure clusters at home using prescribed rescue medications, such as benzodiazepines—particularly in less severe cases, such as focal seizures. As a result, the cohort may disproportionately represent patients with more severe or refractory presentations, or those lacking adequate outpatient support, potentially leading to an unrepresentative sample relative to the broader target population. Another important consideration is that the perceived urgency and clinical approach to seizure clustering may differ substantially between individuals with known epilepsy and those presenting without a prior epilepsy diagnosis. In the former group, seizure clusters may be a recognized and self-managed pattern, whereas in the latter, they are more likely to be viewed as a medical emergency requiring urgent diagnostic evaluation and treatment. This variability in perception may influence the likelihood of ED presentation and, consequently, further limits the generalizability of our findings. The retrospective nature may introduce certain biases, such as information bias due to inconsistent or incomplete data recording, and misclassification of variables such as seizure characteristics, comorbidities, or etiological findings. Another limitation involves the documentation of ASM adherence. Information regarding adherence was based solely on patient or caregiver self-report during the ED evaluation, as no therapy diaries or ASM serum levels measurements were available. As such, the true extent of nonadherence could not be accurately determined. In addition, data on common seizure-precipitating factors—specifically sleep deprivation and physiological stress—were missing in approximately 35% of patients. These are well-recognized seizure triggers, and the lack of consistent documentation further limits our ability to evaluate their contribution to seizure clustering.

This study offers preliminary insights into seizure clusters in emergency settings but is limited by its retrospective design, small sample size, and selection bias. Prospective studies with structured data collection, including validated tools for assessing adherence, lifestyle factors, and seizure triggers, are needed to more accurately characterize the clinical context of seizure clusters.

## Figures and Tables

**Table 1:** Clinical features, diagnostic findings and outcomes by epilepsy history in seizure cluster patients.

	Patients with Epilepsy (*n* = 32)	Patients Without Epilepsy (*n* = 15)
AGE Median (IQR)	47 (IQR 28)	59 (IQR 20)
SEX	M 23	M 9
	F 9	F 0
NUMBER OF SEIZURES DURING CLUSTER Median (IQR)	4 (1.75)	3 (1.5)
SEIZURE TYPE DURING CLUSTER	18	9
GENERALIZED	6	2
FAS	2	1
FIAS	5	0
FAS to BTC	1	3
FIAS to BTC	0	0
UNKNOWN	-	-
PRECIPITATING FACTORS		
Sleep deprivation	0	0
Emotional stress	0	0
Alcohol abuse	5	4
Substance abuse	0	0
MISSED ASM DOSES	4	-
CT FINDINGS		
Normal	13	9
Abnormal	18	5
BRAIN PATHOLOGY		
Remote stroke	9	3
Tumor	2	1
Trauma	3	1
Perinatal injury	4	0
BRAIN LESION LOCALIZATION		
Frontal lobe	8	0
Other localizations	3	6
EEG		
Normal	6	6
Generalized discharges	10	6
Focal discharges	16	3
LABORATORY FINDINGS		
Hyponatremia	2	1
ACUTE INFECTION		
Urinary tract infection	8	3
Pneumonia	5	0
ETHANOL ABUSE	3	5
RESCUE MEDICATION		
Diazepam	6	0
REPEATED SEIZURE CLUSTER	1	0
STATUS EPILEPTICUS	0	0
HOSPITALIZATION	2	3
CLINICAL OUTCOMES		
Need for diazepam in the ED	6	3
Seizure resolution	31	15
Recurrent seizures in ED	1	0
Need for ICU admission	0	0
Changes in ASM regimen	21	-
Introduction of ASM	2	15
Newly diagnosed epilepsy	-	9

**Table 2:** Association of epilepsy with number of seizures during cluster after adjustment for age, comorbidities, and etiology. Multivariable Poisson regression model.

Variable	IRR	Std.Err.	*p* > IzI	IRR 95% CI Lower	IRR 95% CI Upper
Epilepsy	1.05	0.244	0.853	0.65	1.69
Age	0.98 *	0.007	0.004 *	0.97	0.99
Sex	1.1	0.185	0.599	0.77	1.59
UTI	0.79	0.291	0.428	0.45	1.4
Pneumonia	1.52	0.221	0.059	0.98	2.34
Remote stroke	1.11	0.294	0.723	0.62	1.98
Trauma	0.66	0.372	0.259	0.32	1.36
PN brain injury	1.19	0.272	0.517	0.7	2.03
Tumor	0.83	0.491	0.704	0.32	2.17

* increasing age was significantly associated with a lower seizure burden.

**Table 3:** Seizure types during the seizure cluster by epilepsy history. Fisher’s exact test results comparing seizure type frequencies between two groups. After applying both Bonferroni and FDR (Benjamini–Hochberg) corrections, no *p*-values remain statistically significant.

Type of Seizure	Patients WE (*n* = 32)	Patients WOE (*n* = 15)	Fisher’s Exact Test *p*	Bonferroni and FDR Corrections *p*
GTCS	18 (0.56)	9 (0.6)	0.405	1.000
FAS	6 (0.19)	2 (0.13)	0.322	1.000
FIAS	2 (0.06)	1 (0.07)	0.480	1.000
FAS to BTC	5 (0.16)	0	0.052	0.162
FIAS to BTC	1 (0.03)	3 (0.2)	0.026	0.089
UNKNOWN	0	0	-	-

GTCS, generalized tonic–clonic seizure; FAS, focal aware seizure; FIAS, focal impaired awareness seizure; BTC, bilateral tonic–clonic; WE, with epilepsy; WOE, without epilepsy.

**Table 4:** Seizure types reported in patient history versus during seizure clusters. Fisher’s exact test. OR values represent the odds of observing each seizure type during clusters relative to history.

Seizure Type	Types of Seizures in History (*n* = 32)	Seizure Types During Cluster (*n* = 32)	OR	*p*
GTCS	12 (0.37)	18 (0.56)	0.47	0.21
FAS	3 (0.09)	6 (0.19)	0.44	0.47
FIAS	0	2 (0.06)	0	0.49
FAS to BTC	6 (0.19)	5 (0.16)	1.2	1.0
FIAS to BTC	5 (0.16)	1 (0.03)	5.7	0.19
UNKNOWN	6 (0.19)	0	∞	0.02

GTCS, generalized tonic–clonic seizure; FAS, focal aware seizure; FIAS, focal impaired awareness seizure; BTC, bilateral tonic–clonic; WE, with epilepsy; OR, Odds Ratio.

## Data Availability

The data presented in this study are available upon request from the corresponding author due to privacy concerns.

## Abbreviations

The following abbreviations are used in this manuscript:

ACT	acute cluster treatment
BTC	bilateral tonic–clonic
ED	emergency department
EEG	electroencephalograph
EMR	electronic medical records
FAS	focal aware seizure
FIAS	focal impaired awareness seizure
GTCS	generalized tonic–clonic seizure
ICU	intensive care unit
ILAE	International League Against Epilepsy
REST	rapid and early seizure termination
WE	with epilepsy
WOE	without epilepsy
